# Electrospray tandem mass spectrometric analysis of a dimeric conjugate, salvialeriafone and related compounds

**DOI:** 10.1186/1752-153X-6-120

**Published:** 2012-10-18

**Authors:** Syed Ghulam Musharraf, Madiha Goher, Amjad Hussain, M Iqbal Choudhary

**Affiliations:** 1 H.E.J. Research Institute of Chemistry, International Center for Chemical and Biological Sciences, University of Karachi, Karachi 75270, Pakistan; 2 Department of Biochemistry, Faculty of Science, King Abdulaziz University, Jeddah, 21412, Saudi Arabia

**Keywords:** Abietane diterpenoids, Tandem mass spectrometry, *Salvia leriaefolia*, ESI-QqTOF-MS, Salvialeriafone

## Abstract

**Background:**

Electrospray tandem mass spectrometry approach is widely used for the rapid characterization of natural products. This paper describes the gas-phased ESI-MS/MS fragmentation of abietane-type diterpenoids and their novel dimeric conjugate, salvialeriafone (**1**) using both positive and negative ion electrospray ionization quadropole time-of-flight mass spectrometry (ESI-QqTOF-MS/MS) hybrid instrument. Diterpenoids are widely distributed throughout the plant kingdom and posses interesting biological activities.

**Results:**

ESI-QqTOF-MS (positive ion mode) of diterpenoids **1**–**6** under collision-induced dissociation tandem mass spectrometric analysis (CID-MS/MS) showed the characteristic losses of water, carbonmonoxide and propene molecules, while analysis in negative ion mode showed the characteristic losses of water, carbon monoxide, methane molecules and methyl radical. Results demonstrated the differences in the product ions and base peaks due to the differences in the skeleton. A novel dimeric conjugate, salvialeriafone (**1**) showed characteristic fragmentation pattern and was found to be more prone to form radical ions, as compared to monomeric diterpenoids. The fragmentation pathways of characteristic fragments were proposed with the aid of HRESIMS.

**Conclusions:**

Extensive tandem mass spectrometric studies of salvialeriafone (**1**) and related diterpenoids **2**–**6** were conducted and their characteristic fragments were identified. The knowledge of the fragmentation pattern of these diterpenoids will be useful for the characterization of new dimers of this class of compounds.

## Introduction

Diterpenoids constitute a large class of chemically diverse metabolites, widely distributed throughout the plant kingdom with more than 12,000 known examples [1]. Most of the diterpenoids posses diverse biological properties, such as antitumor [2], cytotoxic, antibacterial [3], antiplasmodial [4], leishmanicidal, gastroprotection, molluscicidal [5], antifungal [6], insecticidal [7], rodenticidal [8] and antiproliferative activities. Some of them have effects on cardiovascular and central nervous systems [9].

The genus *Salvia* constitutes the largest of the plant family Labiatea with 900 species wide-spread throughout the world. The genus has yielded various classes of natural products, including the major class of terpenoids, particularly the diterpenoids. Diterpenoids of genus *Salvia* are abietane and neo-clerodane types [10]. More than 400 diterpenoids with different abietane skeletons have been isolated from *Salvia* plants [11]. Diterpenoids from *Salvia* species showed antinflammatory, antidiabetic, ipolipidemic and antiaggregating effects [9]. We have recently isolated salvialeriafone, a dimeric conjugate from *Salvia leriaefolia* which exhibit *in vitro* antiproliferative activity against the human cervical cancer cell line (Hela) [12]. *S. leriaefolia* is used for the treatment of stomach and chronic disorders in Iran.

Many analytical methods including thin-layer chromatography (TLC) [13], high-performance liquid chromatography (HPLC) [14-17] and liquid chromatography–mass spectrometry (LC-MS). [11,18-21] have been used for the analysis of chemical constituents of *Salvia* plants. Tandem mass spectrometric studies of natural products revealed the identification of key fragments which can be helpful for their rapid characterization in the plant extract utilizing LC-MS/MS approach, particularly for the thermally labile compounds. The knowledge of CID-fragmentation pattern of the precursor protonated (or deprotonated) molecule is essentially required prior to the analysis quantification of the desired compounds by LC-MS/MS.

In continuation of our research on the novel characterization and the fragmentation routes of natural product compounds [22-24], we report in this manuscript for the first time the ESI-MS and CID-MS/MS (+ and – modes) of salvialeriafone and its related abietane diterpenoids isolated from *S. leriaefolia*. Knowledge about the characteristic fragments and neutral losses of diterpenoids can be immensely helpful for the rapid identification of these compounds in future phytochemical studies.

## Material and methods

### Standard and reagents

Chemicals and solvents were of analytical and HPLC grades, respectively, and were purchased from Aldrich-Sigma (USA). Deionized water (Milli-Q) was used in the study. Standard diterpenoids were obtained from the Molecular Bank facility at the Dr. Panjwani Center for Molecular Medicine and Drug Research (International Center for Chemical and Biological Sciences), University of Karachi. The isolation procedure and spectroscopic data of the standard diterpenoids has already been reported [12].

## ESI-QqTOF-MS analysis

### Positive ion mode

The diterpenoids were dissolved in methanol, and working dilution was prepared in 1:1 acetonitrile-water containing 0.1% trifluoroacetic acid and analyzed by electrospray ionization (ESI) and collision-induced dissociation (CID), positive ion mode, on QqTOF-MS/MS instrument (QSTAR XL mass spectrometer Applied Biosystem/ MDS Sciex, Darmstadt, Germany) at room temperature. High-purity nitrogen gas was used as the curtain gas and collision gas delivered from Peak Scientific nitrogen generator. The ESI interface conditions were as follows: ion spray capillary voltage of 5500 V, curtain gas flow rate 20 L min^-1^, nebulizer gas flow rate 30 L min^-1^, DP1 60 V, DP2 15 V, and focusing potential of 265 V. The collision energy was swept from 05 to 45 eV for MS/MS analysis. Calibration was performed by using internal calibration process. Sample was introduced into the mass spectrometer using a Harvard syringe pump (Holliston, MA) at a flow rate of 5 μL/ min. MS^2^ Experiment was conducted by selecting the product ion.

### Negative ion mode

The diterpenoids were dissolved in methanol and working dilutions were prepared in 1:1 acetonitrile-water containing 4 mM ammonium acetate (0.2 ng/uL taurochloric acid was used as an internal calibrant) and analyzed by electrospray ionization (ESI) and collision-induced dissociation (CID) negative ion mode on QqTOF-MS/MS instrument (QSTAR XL mass spectrometer Applied Biosystem/ MDS Sciex, Darmstadt, Germany) at room temperature. High-purity nitrogen gas was used as the curtain gas and collision gas delivered from Peak Scientific nitrogen generator. The ESI interface conditions were as follows: ion spray capillary voltage of −4200 V, curtain gas flow rate 20 L min^-1^, nebulizer gas flow rate 25 L min^-1^, DP1 -55 V, DP2 -15 V, and focusing potential of −265 V. The collision energy was swept from 20 to 55 eV for MS/MS analysis. Calibration was performed using internal calibration process. Sample was introduced into the mass spectrometer using a Harvard syringe pump (Holliston, MA) at a flow rate of 5 μL/ min. MS^2^ Experiment was conducted by selecting the product ion.

## Results and discussion

**Figure 1:**
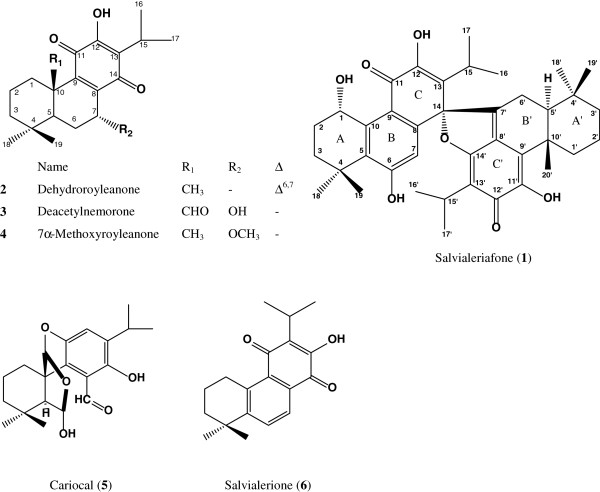
The structures of diterpenoids 1–6.

**Figure 2:**
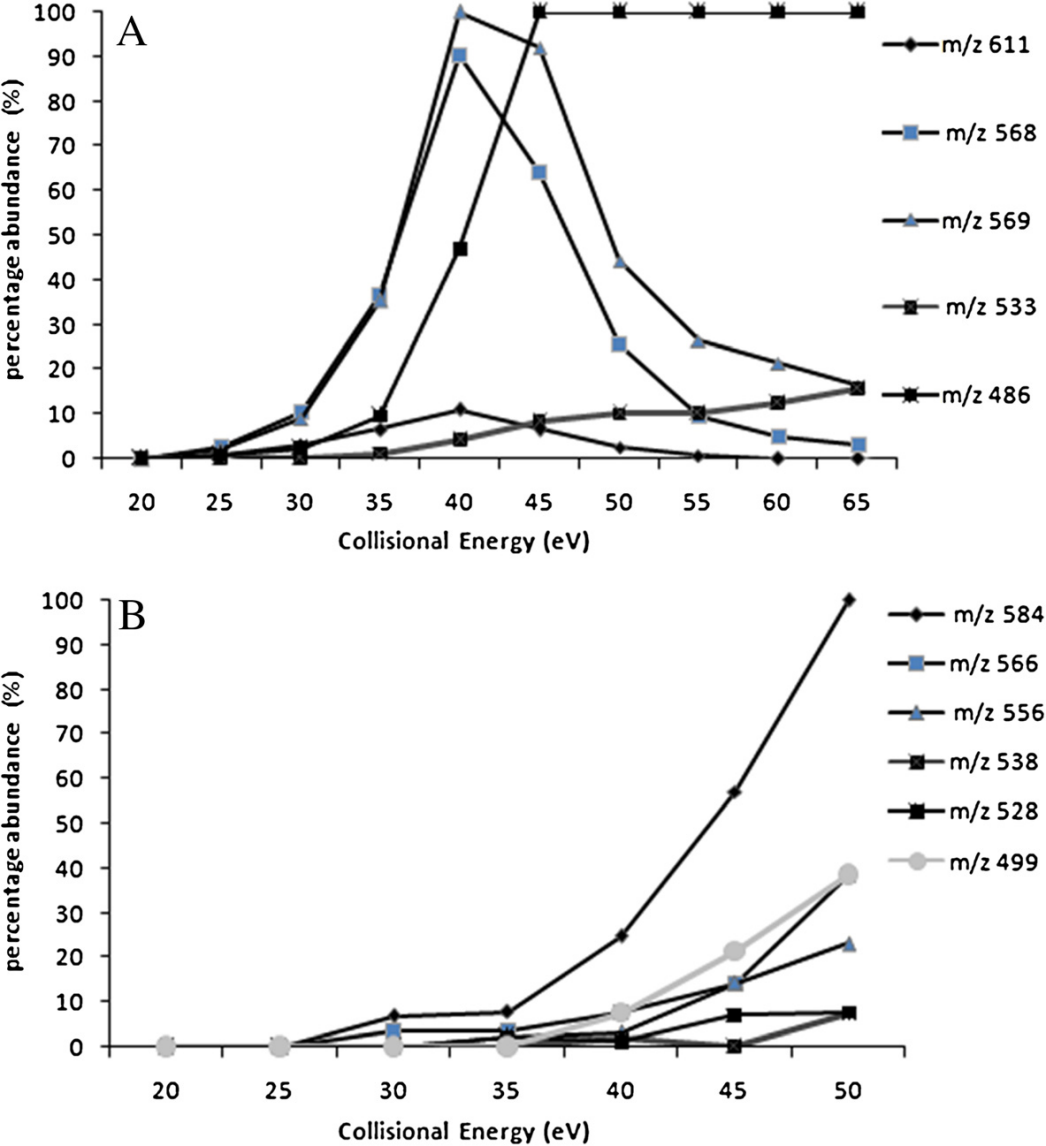
Relative intensity of product ions versus collision energy in the product ion spectra of compound 1 A. in positive ion mode, B. in negative ion mode.

Salvialeriafone (**1**), along with other diterpenoids **2**–**6** (Figure 1), were investigated by the positive and negative ESI-QqTOF-MS analysis. Fragmentation pattern and the product ion abundance were found to be significantly influenced by the variation of collision energy. Therefore MS/MS spectra of all compounds were screened against collision energies, ranging between 20 to 55 eV (with stepping up of 5 eV each time). Relative intensities of product ions of [M+H]^+^ and [M-H]^-^*versus* collision energy ranging were plotted for salvialeriafone (**1)** (Figure 2). It was observed that the product ions formation was at best at collision energy 40 eV (Figure 2A) in positive ion mode, whereas in negative ionization mode the product ions were best formed at the optimum energy collision energy 50 eV (Figure 2B).

### Fragmentation pattern in positive ionization

**Figure 3:**
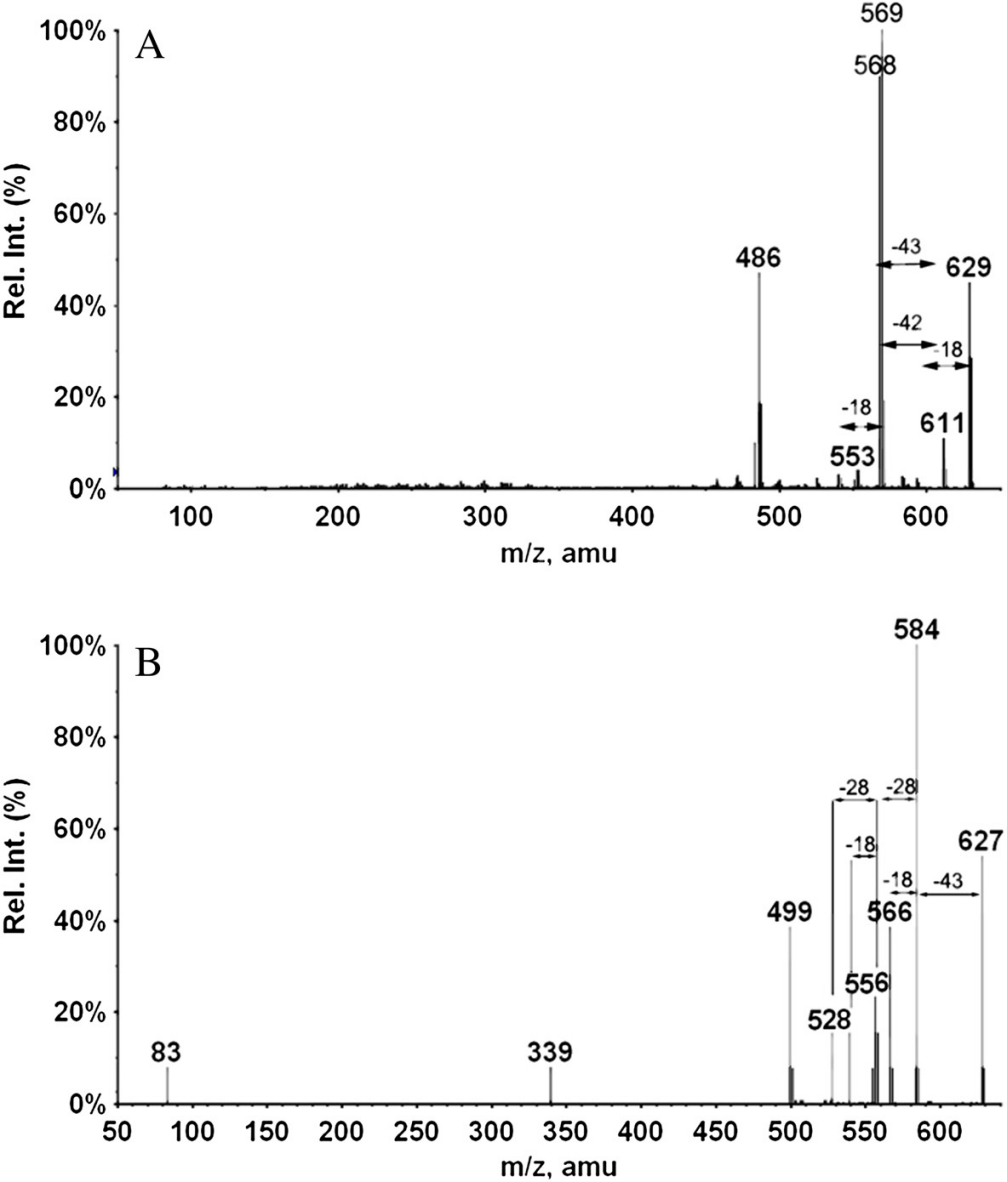
Product ion spectra of Salvialeriafone (1), A. recorded on positive ion mode at collision enegy 40 eV, B. recorded on negative ion mode at collision energy 50 eV.

All diterpenoids produced abundant [M+H]^+^ ions which were selected as the precursor ions to produce MS/MS spectra, but the [M+H]^+^ ion is absent in the MS spectra of compound **5** which gives the dehydrated peak [M+H-H_2_O]^+^, therefore this ion was selected as a precursor ion to produce MS/MS spectra of compound **5**. HRESIMS data of all compounds in positive ion mode is presented in Table 1. All the compounds showed similar fragmentation pattern with minor differences and similar losses of H_2_O, CO, C_3_H_6_ (Table 1), but the extent of fragmentation and optimium collision energy vary due to different substituents and presence of double bonds in the ring. All these diterpenoid **2**–**6** showed intense fragmentation and neutral losses, while compound **1** which is a conjugate of two diterpenoids showed very low fragmentation even at high collision energy. It showed the loss of propene after the water removal as a base peak, i.e. [M+H-H_2_O-C_3_H_6_]^+^ at *m/z* 569.2839 (C_36_H_41_O_6_^+^, calc. 569.2897). The other ions were produced at *m/z* 568.2786 (calc. 568.2819) [M+H-H_2_O-C_3_H_7_]^+^, 611.3297 (calc. 611.3367) [M+H-H_2_O]^+^, 553.2412 (calc. 553.2584) [M+H-C_3_H_6_-CH_4_]^+^. Fragments due to the loss of CO and other consequent lossess did not appear in the MS/MS spectra of compound **1** (Figure 3A).

**Table 1:** Positive ionization HR-ESI-MS data and common neutral losses of diterpenoids 1–6

**S.No.**	**[M+H]**^**+**^	**Exact Mass**	**Observed mass**	**Error** **(ppm)**	**[M+H-H**_**2**_**O]**^**+**^	**[M+H-CO]**^**+**^	**[M+H-C**_**3**_**H**_**6**_**]**^**+**^	**[M+H-2H**_**2**_**O]**^**+**^	**[M+H- C**_**3**_**H**_**7**_**]**^**+.**^	**[M+H-C**_**3**_**H**_**6**_**- H**_**2**_**O]**^**+**^	**[M+H- H**_**2**_**O-CO]**^**+**^	**[M+H-C**_**3**_**H**_**6**_**- H**_**2**_**O-CO]**^**+**^	**Base Peak**
**1**	C_39_H_49_O_7_^+^	629.3472	629.3444	−4.58	611	-	569	-	568 [M+H- H_2_O-C_3_H_7_]^+.^	569	-	-	569 [M+H-C_3_H_6_- H_2_O]^+^
**2**	C_20_H_27_O_3_^+^	315.1965	315.1983	5.49	297	-	273	-	272	255	269	227	245 [M+H-C_3_H_6_-C_2_H_4_]^+^
**3**	C_20_H_27_O_5_^+^	347.1863	347.1871	2.02	329	319	-	311	-	-	301	255	283 [M+H-2H_2_O-CO]^+^
**4**	C_21_H_31_O_4_^+^	347.2227	347.2226	−0.53	315 [M+H- CH_3_OH]^+^	-	-	-	-	297 [M+H- CH_3_OH-H_2_O]^+^	255 [M+H- CH_3_OH-H_2_O-C_3_H_6_]^+^	269 [M+H- CH_3_OH-H_2_O-CO]^+^	269 [M+H- CH_3_OH-H_2_O-CO]^+^
**5**	C_20_H_27_O_5_^+^	347.1863	347.1862	−0.57	329	-		311	-	-	301	259	281 [M+H-2H_2_O-CH_2_O]^+^
**6**	C_19_H_23_O_3_^+^	299.1641	299.1674	10.79	281	-	-	-	256	239	253	211	256 [M+H- C_3_H_7_]^+.^

**Scheme 1:**
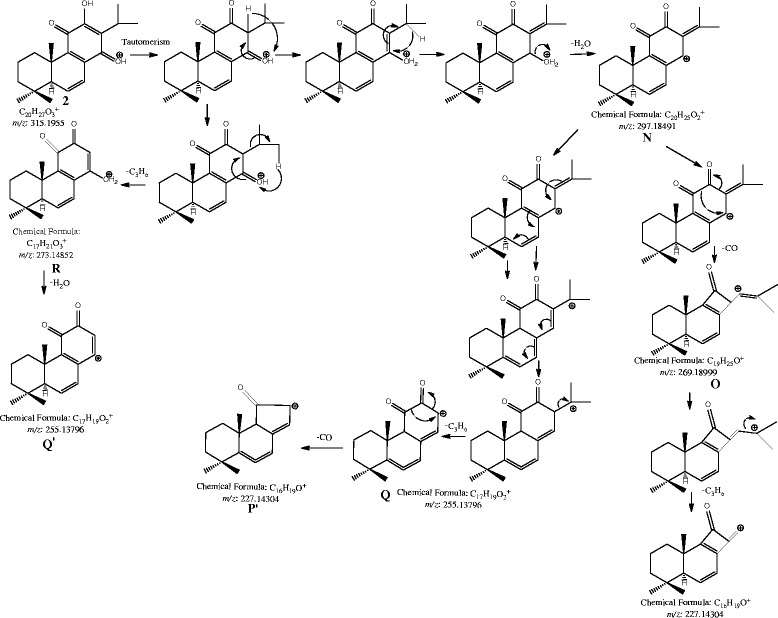
**Proposed fragmentation pathway for the fragments of compound 2 formed due to the removal of CO, C**_**3**_**H**_**6**_**, and H**_**2**_**O through positive ionization.**

The fragmentation pattern of momeric diterpenoids **2**–**6** were studied in order to further validate the neutral lossess and fragmentation pattern of the dimeric conjugate, salvialeriafone (**1**). All diterpenoids showed the removal of water molecule, i.e. [M+H-H_2_O]^+^ except compound **4** which showed the removal of methanol due to the presence of methoxy group followed by the loss of water molecule. A loss of water molecule followed by the loss of CO was observed in compounds **2**, **3**, **5**, and **6**, while compounds having OH group in ring B (compounds **3** and **5**) showed the loss of two water molecules, i.e. [M+H-2H_2_O]^+^. All analyzed compounds showed the removal of propene moiety directly from the [M+H]^+^ and/or after the removal of H_2_O and CO groups. These compounds also showed the neutral lossess of ethene and butane molecules. Compounds **3** and **5** having same molecular formula but difference of skeleton in ring B produced differentiated base peak and some other abundant peaks (Table 1). Compound **3** showed the base peak at *m/z* 283 [M+H-2H_2_O-CO]^+^, while compound **5** showed the base peak at *m/z* 281 [M+H-2H_2_O-CH_2_O]^+^ and compound **5** also showed the intense fragments at *m/z* 217 and 205, while the peak at *m/z* 319 [M+H-CO]^+^ is absent in compound **5**. Like compound **1**, compounds **2** and **6** also showed the presence of abundant peaks of radical cations due to the loss of propyl radical. Unlike monomeric diterpenoids the product ion spectra of compound **1** was very simple, having only a few peaks in the range of ≥ *m/z* 486. However, being a dimer, compound **1** possess two isopropyl and four hydroxyl substituents, but showed the loss of substituents equivalent to the loss which are derived from a monomeric diterpenoid unit. Fragmentation pathway for the fragments formed due to the removal of CO, C_3_H_6_, and H_2_O has also proposed as and shown in Scheme 1 (compound **2** is taken as a representive of monomeric diterpenoids).

### Fragmentation pattern in negative ionization

All diterpenoids **1**–**6** produced abundant [M-H]^-^ ions, which were selected as the precursor ions to produce MS/MS spectra. The QTOF-MS/MS the low-energy collision induced dissociation tandem mass spectrometry experiments (CID-MS/MS) were optimized and developed through ramping collision voltage to induce more product ions and the optimum collision energy for recording product ion spectra of diterpenoids in negative ion mode is ranging from 30 to 45 eV HRESIMS data of all compounds in negative ion mode is presented in Table 2. In negative ion mode, all the compounds showed different fragmentation pattern due to different substituents and the presence of double bonds in the ring. These compounds showed the common losses of H_2_O, CO, CH_4_, and CH_3_ radical, similar to positive ion mode, except the loss of CH_4_, and CH_3_ radical Table 2.

**Table 2:** Negative ionization HR-ESI-MS data and common neutral losses of diterpenoids 1–6

**S.No.**	**[M-H]**^**-**^	**Exact Mass**	**Observed mass**	**Error (ppm)**	**[M+H-H**_**2**_**O]**^**-**^	**[M+H-CO]**^**-**^	**[M+H-CH**_**4**_**]**^**-**^	**[M+H-CH**_**3**_**]**^**-**^	**[M+H- C**_**3**_**H**_**7**_**]**^**-.**^	**[M+H-C**_**3**_**H**_**7**_**- CO]**^**-.**^	**[M+H- H**_**2**_**O-CO]**^**-**^	**[M+H-CH**_**4**_**-CO]**^**-**^	**Base Peak**
**1**	C_39_H_49_O_7_^+^	627.3327	627.3372	7.1286	-	-	-	-	584	556	538 [M+H-C_3_H_7_- CO-H_2_O]^-.^	-	584 [M+H-C_3_H_7_]^-.^
**2**	C_20_H_27_O_3_^+^	313.1809	313.1789	−6.4452	-	285	297	298	-	-	-	269	285 [M+H-CO]^-^
**3**	C_20_H_27_O_5_^+^	345.1707	345.1694	−3.9046	327	317	-	-	-	-	299	-	317 [M+H-CO]^-^
**4**	C_21_H_31_O_4_^+^	345.2071	345.2075	1.0622	315 [M+H-CH_2_O]^-^	-	-	330	-	-	287 [M+H-CH_2_O-CO]^-^	-	
**5**	C_20_H_27_O_5_^+^	345.1707	345.1698	−2.7458	327	317	-	-	-	-	-	-	179
**6**	C_19_H_23_O_3_^+^	297.1496	297.1512	5.3227	-	269	281	282	-	-	-	253	269 [M+H-CO]^-^

Compound **1**, which is a dimer of two diterpenoids, showed the loss of propene radical as a base peak i.e. [M-H-C_3_H_7_]^-**.**^ at *m/z* 584.2777 corresponding to the molecular formula C_36_H_40_O_7_ (calc. 584.2779). The other ions were found to be at *m/z* 566 [M-H-C_3_H_7_-H_2_O]^-^, 556 [M-H-C_3_H_7_-CO]^-^, 538 [M-H-C_3_H_7_-H_2_O-CO]^-^ and 528 [M-H-C_3_H_7_-2CO]^-^ (Figure 3B). But the lossess of methane and methyl radical were not appeared in the product ion spectra of compound **1**, in comparision with other monomeric diterpenoids.

**Scheme 2:**
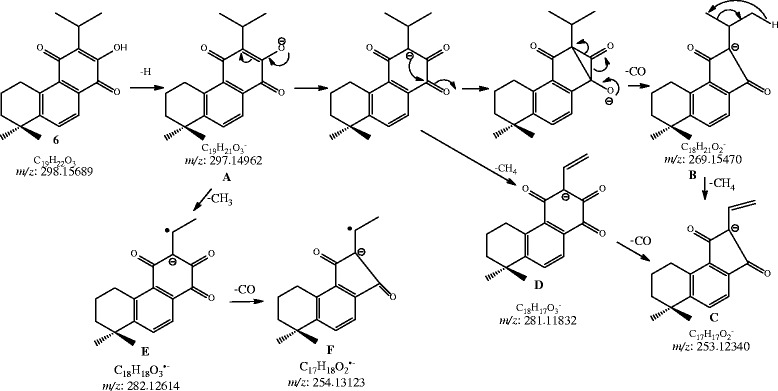
**Proposed fragmentation pathway for the fragments of compound 6 formed due to the removal of CO, CH**_**4**_**, and CH**_**3**_**through negative ionization.**

In monomeric compound **2**, the removal of CO, i.e. [M-H-CO]^-^ yielded a base peak at *m/z* 285, corresponding to the formula C_19_H_25_O_2_ (observed 285.1923, calc. 285.1923). The other abundant ions were *m/z* 297 [M-H-CH_4_]^-^, 269 [M-H-CH_4_-CO]^-^ and 227 [M-H-CH_4_-CO-C_3_H_6_]^-^, while the minor ions at *m/z* 298 [M-H-CH_3_]^-^, 270 [M-H-CH_3_-CO]^-^ and 148 [M-H-CH_3_-CO]^-^ were produced due to the loss of CH_3_ radical. Compounds **3** and **5**, having same molecular formula, showed similar losses of H_2_O, CO, CO_2_ from the precursor ions. The ions are produced at *m/z* 327 [M-H-H_2_O]^-^, 301 [M-H-CO_2_]^-^, 317 [M-H-CO]^-^ , 299 [M-H-CO-H_2_O]^-^ and 271 [M-H-2CO-H_2_O]^-^. As the compound **5** have different skeleton in which the ring B has been modified it produce a base peak at *m/z* 179.0734 due to the fragment ion C_10_H_11_O_3_^-^ (calc. 179.0713), while in compound **3** the base peak was appeared at *m/z* 317 [M-H-CO]^-^. Compound **6** yielded the base peak at *m/z* 269 [M-H-CO]^-^ and the other ions were found to be at *m/z* 281 [M-H-CH_4_]^-^, 282 [M-H-CH_3_]^-.^, 253 [M-H-CO-CH_4_]^-^ and 254 [M-H-CH_3_-CO]^-^. The product ion spectra of compound **1** in negative ionization was very simple with only a few peaks in mass range of ≥ *m/z* 499. Unlike compound **1**, compounds **2**–**6** did not show the lossess of propene and propyl radical in negative ionization. Fragmentation pathway for the fragments formed due to the removal of CO, CH_4_, and CH_3_ through negative ionization has also been proposed and shown in Scheme 2 (compound **6** is taken as a representive of monomeric diterpenoids).

## Conclusion

In conclusion, fragmentation pattern of six abietane-type diterpenoids and one novel dimeric conjugate diterpenoid, salvialeriafone (**1)** have been studied by using ESI-QqTOF-MS/MS in both positive and negative ionization mode. It has been observed that many characteristic neutral losses and formation of key fragment ions can provide important structural information about the basic skeleton of abietane-type diterpenoids having dimeric linkages. The dimeric conjugate showed somewhat different pattern and less fragmentation as compared to monomeric diterpenoid analogue. The knowledge of fragmentation pattern is immensely helpful for the rapid characterization of abietane-type diterpenoids through liquid chromatography coupled with mass spectrometry in complex mixtures such as plant extracts or herbal formulations by utilizing their analytical amount.

## Competing interests

Authors declare that they have no competing interests.

## Authors’ contributions

SGM: Supervised the whole study and participated in manuscript preparation. MG: Involved in performing experimental and manuscript preparation. AH: Participated in purification of standard compounds. MIC: Involved in supervision of isolation of compounds **1**–**6**, useful discussions and participated in manuscript preparation. All authors read and approved the final manuscript.
